# PPG2ECGps: An End-to-End Subject-Specific Deep Neural Network Model for Electrocardiogram Reconstruction from Photoplethysmography Signals without Pulse Arrival Time Adjustments

**DOI:** 10.3390/bioengineering10060630

**Published:** 2023-05-23

**Authors:** Qunfeng Tang, Zhencheng Chen, Rabab Ward, Carlo Menon, Mohamed Elgendi

**Affiliations:** 1 School of Life and Environmental Sciences, Guilin University of Electronic Technology, Guilin 541004, China; 2 Department of Electrical and Computer Engineering, University of British Columbia, Vancouver, BC V6T 1Z1, Canada; 3 Biomedical and Mobile Health Technology Lab, Department of Health Sciences and Technology, ETH Zurich, 8008 Zurich, Switzerland

**Keywords:** electrocardiogram construction, remote monitoring, digital health, AI in healthcare, photoplethysmography

## Abstract

Electrocardiograms (ECGs) provide crucial information for evaluating a patient’s cardiovascular health; however, they are not always easily accessible. Photoplethysmography (PPG), a technology commonly used in wearable devices such as smartwatches, has shown promise for constructing ECGs. Several methods have been proposed for ECG reconstruction using PPG signals, but some require signal alignment during the training phase, which is not feasible in real-life settings where ECG signals are not collected at the same time as PPG signals. To address this challenge, we introduce PPG2ECGps, an end-to-end, patient-specific deep-learning neural network utilizing the W-Net architecture. This novel approach enables direct ECG signal reconstruction from PPG signals, eliminating the need for signal alignment. Our experiments show that the proposed model achieves mean values of 0.977 mV for Pearson’s correlation coefficient, 0.037 mV for the root mean square error, and 0.010 mV for the normalized dynamic time-warped distance when comparing reconstructed ECGs to reference ECGs from a dataset of 500 records. As PPG signals are more accessible than ECG signals, our proposed model has significant potential to improve patient monitoring and diagnosis in healthcare settings via wearable devices.

## 1. Introduction

Electrocardiograms (ECGs) are widely used to diagnose many cardiovascular diseases. The continuous monitoring of ECGs is becoming increasingly important in personal healthcare as the world’s population increases and ages. An ECG detects the heart’s electrophysiological activity through electrodes placed on the skin, providing information about the cardiovascular system. However, measuring the ECG with standard 12-lead ECG equipment limits the patient’s activities, which makes it inconvenient. Moreover, placing multiple electrodes at different locations can cause skin irritation and discomfort during the ECG recording. Photoplethysmography (PPG), another signal that reflects the cardiovascular system’s state, has attracted widespread attention in the last 10 years due to its advantages of easy acquisition, small sensor sizes, and non-invasiveness. PPG is an optically acquired signal that can be used to detect changes in blood volume in the microvascular beds of tissues. The duration, amplitude, and morphological features of the PPG waveform can be translated into physiological parameters, such as oxygen saturation [1], blood pressure [2], and cardiac output [3], among others. Wearable devices equipped with PPG sensors, such as wristbands and finger probes [4,5], are commonly used. These PPG-based devices are generally smaller, less expensive, more comfortable, and easier to use than ECG devices. Additionally, patients can use them in their daily lives with minimal expertise. However, although PPGs are frequently used for healthcare monitoring, ECGs remain the standard and fundamental measurements for medical diagnoses, with a wealth of supporting literature and research. Clinicians still rely on ECGs rather than PPGs for diagnoses in clinical settings. Therefore, if PPGs can be used to reconstruct ECGs, it would be possible to take advantage of the easy access afforded by PPGs and use the rich available knowledge for ECGs to diagnose the condition.

The reconstruction of ECG signals using PPGs is feasible. From a physiological perspective, an ECG signal is a collection of electrophysiological cardiac motion signals, while PPGs reflect cardiac mechanical motions. The electrophysiological activity and mechanical movement of the heart are linked through excitation–contraction coupling [6]. Signal analysis studies have shown that PPG and ECG signals are highly correlated in cycle duration [7]. For example, arrhythmia can be detected in PPG when it appears in the ECG [8,9]. Moreover, the ECG parameters can be estimated by the PPG features [10]. The heart and the entire cardiovascular system can be simplified as black boxes. The ECG signal is used as the input of this black box and the PPG signal is used as the output. In this case, using the PPG to reconstruct the ECG can be seen as the inverse problem of finding the transfer function of this black box.

**Figure 1:**
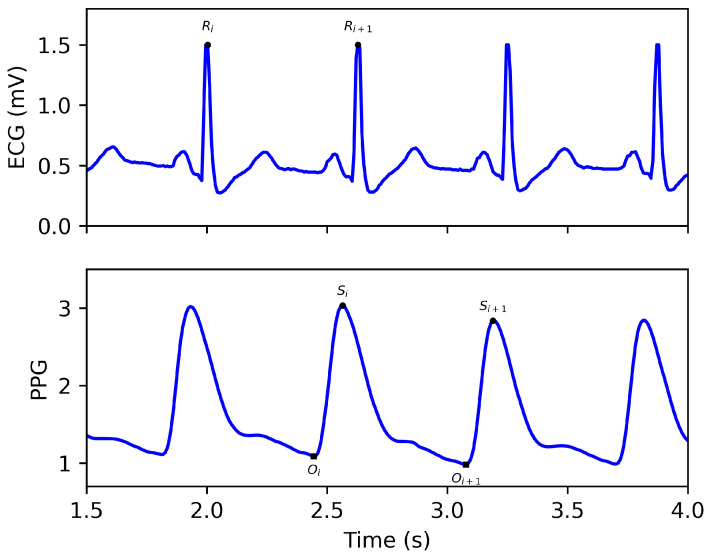
A synchronized ECG and PPG signal. $R_{i}$ in ECG refers to the *i*th R peak. $O_{i}$ and $S_{i}$ refer to the *i*th onset and systolic peak in the PPG signal, respectively.

Figure 1 shows a pair of synchronized ECG and PPG signals. The R peak is the main morphology feature in the ECG waveform [11,12]. The onset and systolic peaks are the two main features of the PPG [13,14]. An expanded discussion of PPG features can be found here [15]. For the *i*th cycle, the R wave in the ECG represents the depolarization of the ventricle, causing the ventricle to contract and the heart to eject blood. It takes a while for the blood to be transmitted to the site where the PPG is detected (usually fingertips, wrists, and earlobes) [16]. Therefore, the PPG’s onset and the systolic wave’s peak occur later than the ECG’s R wave. The time interval between the R wave of the ECG and the onset of the PPG is referred to as the pulse arrival time (PAT) [17]. The PAT is within 2 s and varies based on the detection site of the PPG, blood pressure, etc. Therefore, when reconstructing the ECG, it is necessary to establish the PAT and the relationship between the PPG and the ECG waveforms.

Several studies have attempted to reconstruct ECG signals from PPG signals using various techniques. Two studies employed the DCT method [18] and cross-domain joint dictionary learning [19] to reconstruct ECG signals beat-by-beat. These methods involved aligning the onset in the PPG with the R peak in the ECG to remove the PAT. Then, the aligned ECG and PPG signals were segmented into cycles, and a mapping from the pulse wave pulse to the ECG cycle was established. The ECG signal was reconstructed from the PPG based on this mapping. The Bi-LSTM model [20] used in our previous study generated the ECG segment-by-segment without requiring cycle segmentation; however, it did require an alignment step. Nevertheless, these techniques demanded an alignment step in signal preprocessing, which involves the requirement of the ECG as a reference to align PPG signals, making it impractical since the primary objective of the ECG reconstruction from the PPG is to avoid the necessity of the ECG. Furthermore, while the RR intervals in the ECG were highly correlated with the onset-to-onset interval in the PPG [7], they were not the same, and certain diseases could make the RR interval differ from the systolic peak-to-systolic peak interval [21]. Three studies [22,23,24] using deep neural networks to reconstruct ECGs from PPGs did not require alignment steps in preprocessing. Two studies focused on the heart rate destination without emphasizing the quality of the ECG waveform, and one study [23] was not a subject-specific model. Therefore, this paper aims to develop a subject-specific model that can reconstruct ECG signals that are highly similar to real ECG signals, without the need for calculating or adjusting for the PAT.

## 2. Materials and Methods

**Figure 2:**
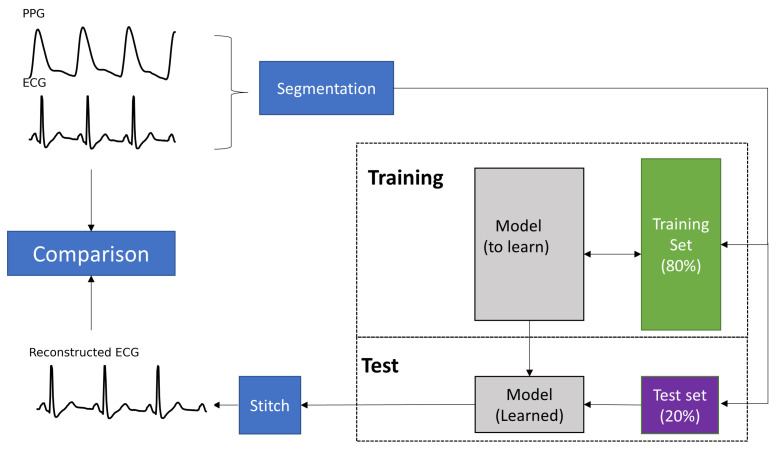
Flowchart of the proposed method. The synchronized ECG and PPG signals are segmented into segments with 1024 samples. The first 80% of segments are used for training, and the last 20% are used for the test. The output of the learned model involves segments with 1024 ECG samples, the stitch step is used to stitch a segment to generate an ECG signal.

This section describes the dataset used, the preprocessing of the ECG and PPG signals, the deep neural network architecture, and the model performance evaluation. Figure 2 shows the flowchart of the proposed method.

It is important to note that all of the codes were implemented in Python 3.9, and the neural network was implemented based on the TensorFlow 2.8.0 end-to-end open-source platform for machine learning. NVIDIA GeForce RTX 3060 Ti and Intel Core i7-11700 @ 2.50 GHz were the hardware used to run the model.

### 2.1. Dataset

The present study utilized the cuffless blood pressure estimation dataset [25], which was compiled by Kachuee et al. from the MIMIC II database [26]. This dataset comprises a total of 12,000 records with varying durations, wherein each record includes photoplethysmogram (PPG), arterial blood pressure (ABP), and lead II electrocardiogram (ECG) signals sampled at a rate of 125 Hz. The current investigation included only the first 500 records in the dataset with signals exceeding 8 min in length, wherein solely PPG and lead II ECG signals were analyzed. It remains unclear if the 500 recordings were acquired from different subjects, yet each recording was treated as a unique subject in this study.

### 2.2. Preprocessing

The raw signals were formed of pairs of long-term synchronized ECG and PPG signals. The ECG and PPG signals were segmented with 1024 samples (equivalent to 8.192 s based on the sampling frequency) and a stride size of 256 samples, which means that there was an overlap of 768 samples between every 2 consecutive segments. A segment was discarded if the final segment was less than 1024 samples. The dataset used in our study contains records of varying lengths, which can affect the number of signal segments present in each record. It is noteworthy that all recordings exceeded 8 min in length but were not of equal duration. In order to address this variation, a uniform criterion was applied to define the training and test sets. In particular, the first 80% of each recording was used for training, whereas the remaining 20% of each recording was used for testing.

### 2.3. Model Architecture

**Figure 3:**
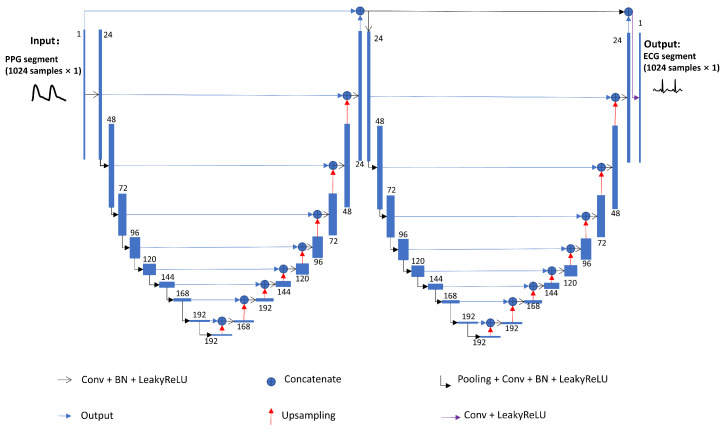
Architecture of the proposed neural network. The ‘Conv’, ‘Pooling’, and ‘Upsampling’ denote a one-dimensional convolution layer, a max-pooling layer, and upsampling in the time direction by 2, respectively. ‘LeakyReLU’ refers to the activation function of the corresponding convolution layer. ‘BN’ denotes a 1D batch normalization layer.

The proposed algorithm’s name, PPG2ECGps, is reflective of its focus on using PPG signals to reconstruct ECG signals, with the ‘ps’ suffix indicating its patient-specific nature. The architecture of the PPG2ECGps algorithm is illustrated in Figure 3. A similar neural network was used to reconstruct an arterial blood pressure signal from PPG [27]. In Figure 3, the terms ‘Conv’, ‘Pooling’, and ‘Upsampling’ denote a one-dimensional (1D) convolution layer, a max-pooling layer, and upsampling by 2 in the time direction, respectively. ‘LeakyReLU’ refers to the activation function of the corresponding convolution layer. ‘BN’ denotes a 1D batch normalization layer. The slope of the ‘LeakyReLU’ activation is set to 0.1.

The proposed W-Net was composed of two U-blocks. The output of the first U-block was concatenated with the input of the whole architecture to become the input of the second U-block. The U-block was inspired by the wave U-Net [28]. The wave U-Net is a full convolution neural network; it was first used in audio source separation. Moreover, its variants have been used to reconstruct ABP signals from the PPG [29]. In the proposed W-Net, the convolution layers are followed by batch normalization and the ‘LeakyReLU’ activation function. The filter size of the convolution layer is set to 15. The last convolution layer of W-Net is directly activated by ‘LeakyReLU’, and the filter size is set to 1. In studies on image analysis, the method composed of two U-blocks was proven to perform better than the method using one U-block [30,31].

### 2.4. Stitching the Reconstructed ECG Segments

The output of the neural network comprises 8.192 s (1024 samples) of reconstructed ECG segments, which need to be stitched together to form a continuous ECG recording. This stitching is performed in a loop fashion, where the stitched ECG signal “S3” is obtained as a result of stitching the first and second segments, “S1” and “S2”, respectively. As there is a 75% overlap (768 samples) between the two consecutive segments, the last 768 samples of “S1” are discarded to obtain the final stitched ECG segment as a result of combining S1 and S2.

### 2.5. Training Options

The neural network used an Adam optimizer. To ensure that the neural network achieves optimal performance without overfitting, it is important to set a stopping criterion when training the model. In our study, we set the maximum number of training epochs to 500 and used a batch size of 128 pairs of segments.

Finding the right balance between the training speed and model performance is crucial. To achieve this, we propose a simple yet effective method: starting with a high learning rate to expedite training and gradually reducing it to enhance performance. Research has shown that this technique, known as learning rate attenuation, can improve the performance of popular neural networks [32,33]. In our study, we set the initial learning rate to 0.001 and then decayed it by 0.1 every 800 steps during training. This approach helped us achieve a better balance between the training speed and model performance, resulting in more accurate and efficient results. The loss function used in this study is defined as follows:

(1) Loss=mal+mse+(1−|r|),

where

(2) $$\left\{\begin{array}{c} mal=\max_{1\leq i\leq l}\left(|ECG_{ref}\left(i\right)-ECG_{rec}\left(i\right)|\right),\\ mse=\frac{1}{l}\sum_{i=1}^{l}{\left(ECG_{ref}\left(i\right)-ECG_{rec}\left(i\right)\right)}^{2},\\ r=\frac{\sum_{i=1}^{l}\left(ECG_{ref}\left(i\right)-\bar{ECG_{ref}}\right)\sum_{i=1}^{l}\left(ECG_{rec}\left(i\right)-\bar{ECG_{rec}}\right)}{\sqrt{\sum_{i=1}^{l}{\left(ECG_{ref}\left(i\right)-\bar{ECG_{ref}}\right)}^{2}}\sqrt{\sum_{i=1}^{l}{\left(ECG_{rec}\left(i\right)-\bar{ECG_{rec}}\right)}^{2}}}, \end{array}\right.$$

note that mal, mse, and *r* refer to maximal absolute loss (MAL), mean squared error, and Pearson’s correlation coefficient (*r*) [34], respectively. $ECG_{ref}\left(i\right)$ and $ECG_{rec}\left(i\right)$ are the *i*th sample points of the reference ECG and reconstructed ECG, respectively. The variables *l*, $\bar{ECG_{ref}}$, and $\bar{ECG_{rec}}$ are the length of the test ECG signal, as well as the averages of the sample value of the reference ECG and the reconstruction ECG, respectively.

In the loss function, *mse* and *r* restrict the consistency between the reconstructed ECG value and the reference ECG waveform, respectively. Moreover, *r* is usually used to measure the linear correlation between signals, and its value is in the range of [−1, 1]. An *r* value of ±1 indicates the strongest correlation, while 0 indicates the weakest correlation. Furthermore, *mse* and *r* ensure global similarity. However, the duration of the R wave is short, and the values of the sampling points in it change rapidly. Thus, the *mse* and *r* have limitations in this event. In this case, the MAL was introduced to ensure that the R wave of the reconstructed ECG matches that of the reference ECG.

### 2.6. Performance Evaluation

Three measures were used to evaluate the performance of the reference ECG signal and the reconstructed ECG signal in the proposed model: root mean squared error (RMSE), Pearson’s correlation coefficient (*r*), and the normalized dynamic time warping (DTW) distance.

**Root mean square error (RMSE):** In machine learning, RMSE is commonly used to measure the model’s estimated and observed values. The formula of RMSE is as follows:

(3) $$RMSE=\sqrt{\frac{1}{l}\sum_{i=1}^{l}{\left(ECG_{ref}\left(i\right)-ECG_{rec}\left(i\right)\right)}^{2}}.$$

**Normalized dynamic time warping distance.** DTW can measure the similarity between two time series with potentially different velocities [35]. Our previous study [20] found that there may be a phase error (several samples) between the reconstructed ECG and the reference ECG. Therefore, in the present study, we introduce the DTW distance to evaluate the similarity between the reconstructed and reference ECGs.

The steps to calculate the DTW are as follows:

- Calculate the Euclidean distance between every sample from the reconstructed ECG and every sample from the reference ECG. For the *i*th sample of reconstructed ECG and the *j*th sample of the reference ECG, the Euclidean distance is defined as follows:
  (4) $$d_{ij}=\sqrt{{\left(ECG_{ref}\left(i\right)-ECG_{rec}\left(j\right)\right)}^{2}+{\left(i-j\right)}^{2}},$$
  where *i* and *j* are the indices of the samples of the reference ECG and reconstructed ECG, respectively. Suppose that the number of samples of the reference ECG signal is *N*. This step will create an N×N matrix *A*.
- Look for paths in matrix *A* that start at $d_{11}$ and end at $d_{NN}$. For any point on the path, the next point can only be one of its right, upper, or upper-right corners. Calculate the sum of the distance along the paths. The minimum sum along the paths is the DTW distance, and this path is defined as the warping path.

The smaller the DTW distance, the more similar the reference and reconstructed ECG. However, the DTW distance increases with the length of the reference and reconstructed ECG. To better evaluate the similarity of the two time series, the DTW distance was normalized in this study by dividing the DTW distance by the sum of the length of the reference and reconstructed ECG.

The formula is as follows:

(5) $$\bar{d}=\frac{d_{11}+\cdots +d_{NN}}{2N},$$

where *N* is the length of the reference ECG signal, and the subscript of *d* is consistent with the warping path.

## 3. Results

**Figure 4:**
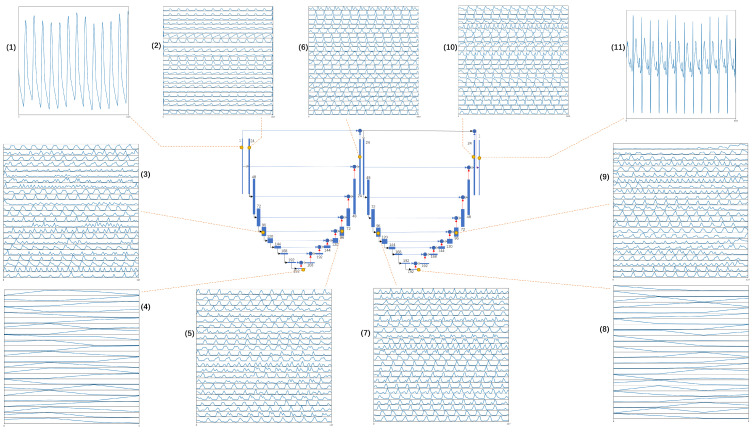
Maps of learned features of the proposed W-Net architecture. Graphs (1) and (11) are the input and the output, respectively. Graphs (2)–(10) show the first 24 of all of the learned features in the corresponding feature maps.

A neural network can be considered as a black box that takes the input through a series of transformations and generates the output. Feature visualization transforms the features learned by the neural network into information that can be understood. Figure 4 shows some of the features learned by the proposed W-Net. For example, (1) and (11) are the input and output, respectively. As seen in (2), (3), and (4), the W-Net learns the time domain features of the PPG waveforms, such as systolic and diastolic peaks, as reported in [13]. Then, as the PPG signal was pooling, W-Net paid more attention to the inter-period features. Similarly, by comparing (8), (9), and (10), the feature map shows additional time-domain features of the ECG signal as the signal is upsampled. For (5), (6), and (7), it is not easy to understand which features are extracted. However, it can be seen that these features appear periodically. This reflects the high correlation between PPG and ECG in terms of beats.

**Figure 5:**
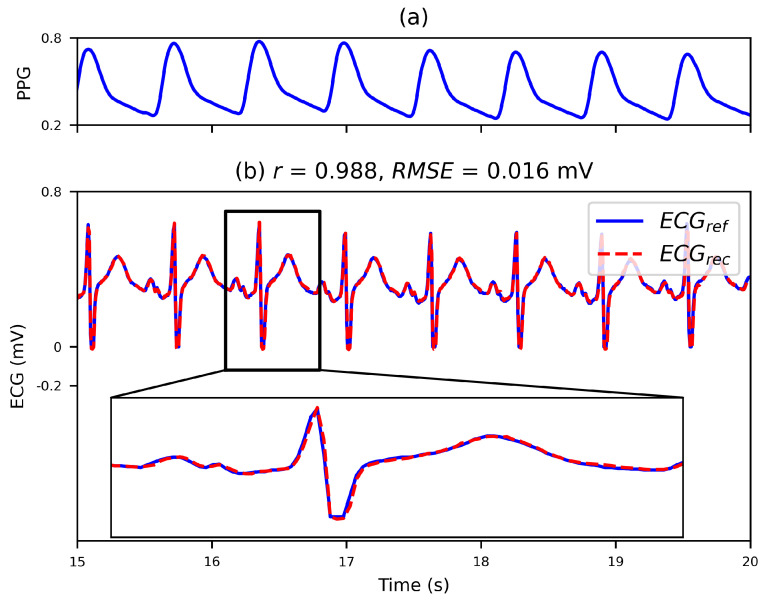
Demonstration of the reconstruction of the ECG waveform; ‘*r*’ and ‘RMSE’ stand for Pearson’s correlation coefficient and the relative mean squared error, respectively. (**a**) The PPG used to reconstruct the ECG. (**b**) Comparison of the reference ECG and the reconstructed ECG.

Figure 5 shows a segment of the reconstructed ECG waveform. Figure 5a shows the PPG, which is the input to the model. Figure 5b shows the reconstructed and reference ECGs. As seen, the reconstructed ECG is very similar to the reference ECG, with no phase errors and little difference in values. For the reconstructed ECG and the reference ECG, Pearson’s *r* reached 0.988, while the *RMSE* was only 0.016 mV.

**Figure 6:**
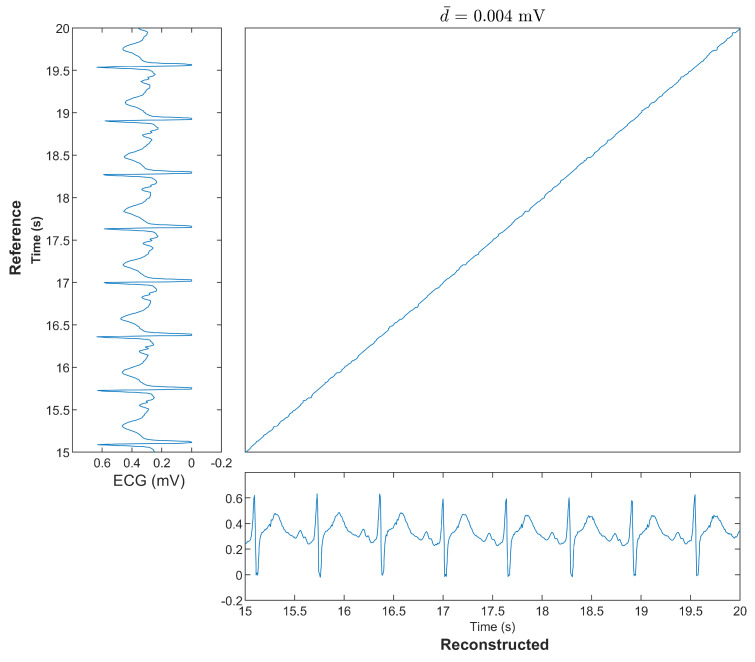
A segment of the optimal DTW warping path for the reference ECG and reconstructed ECG. The $\bar{d}$ stands for the normalized DTW distance.

Figure 6 shows the DTW warping path of the reconstructed ECG and the reference ECG in the same segment, as shown in Figure 5. The DTW warping path provides another view to evaluate the similarities between the reconstructed ECG and reference ECG. The warping path is nearly a straight line. A straighter warping path means less warping is required to map the reconstructed ECG and reference ECG; this means there is more similarity between these two signals. The warping path looks similar to a straight line in Figure 6. The normalized DTW distance between the reconstructed ECG and reference ECG is 0.004 mV.

In our previous study [20], we found that there may be a small phase error between the reconstructed ECG and the reference ECG, and this phase error affects the results in Pearson’s correlation coefficient and *RMSE*. Therefore, we introduced cross-correlation to determine the time delay between the reconstructed and reference ECGs. Then, these two signals were aligned by delaying the earliest one. This step can remove the effects of phase errors. To discuss the effects of the phase errors, this paper presents two experiments that were performed. In Experiment I, the three measures were directly used on the reconstructed ECG and reference ECG. In Experiment II, the reconstructed ECG and the reference ECG were first aligned based on cross-correlation, and then the model’s performance was evaluated. The results are shown in Table 1. No significant difference was found between the two experimental results. Consequently, the ECG reconstructed using this model is essentially free of phase errors. Moreover, in Experiment I, Pearson’s *r* and *RMSE* were, on average, 0.977 and 0.037 mV, respectively. Thus, the reconstructed ECGs are highly correlated with the reference ECGs.

## 4. Discussion

We propose the PPG2ECGps, which is a method that uses the W-Net neural network architecture to reconstruct ECG signals from PPG signals. A comparison of the proposed model with existing methods is shown in Table 2. The main difference between the proposed model and existing methods is that the proposed model does not need to align the PPG signal with the ECG signal.

These signals are not in alignment because there is a delay in the time from when the blood is transmitted from the heart to the site where the PPG is detected (usually fingertips, wrists, and earlobes) [16]. Therefore, the onset of the PPG and the peak of the systolic wave occurs later than the R wave of the ECG. To eliminate the PATs, existing methods align the PPG and ECG signals. The alignment step requires extracting certain handcrafted features (such as the R waves in ECG and the systolic peaks and onsets in PPG).

Although handcrafted feature extraction algorithms have been used to extract important features from ECG signals, they can introduce errors that negatively impact the performance of the overall model. To address this issue, we propose a new W-Net neural network architecture based on convolutional neural networks (CNNs) that can automate the feature extraction process, leading to better results [38]. Our approach uses PPG signals as they are, without any adjustment or counting for the PAT. Additionally, our method is subject-specific, meaning that the neural network can learn the unique characteristics of each subject during the training phase, leading to even more accurate and personalized results.

In comparison with our previous study on reconstructing arterial blood pressure signals [27], this study modified the activation function of the last convolution layer from Tanh to ‘LeakyReLU’. This modification allows the neural network to produce signals with values greater than 1. In clinical settings, the amplitude of the R wave in a normal ECG signal may exceed 1 mV [39]. The use of ‘LeakyReLU’ as the activation function eliminates the need to normalize the reconstructed ECG signal to the range [−1, 1], thereby improving the model’s robustness.

Note that when choosing activation functions for deep neural networks [40,41,42], some commonly used functions, such as “sigmoid” are not suitable due to slow convergence and the problem of gradient disappearance. Other functions, such as “Tanh”, converge faster but still suffer from gradient disappearance. The “ReLU” activation function is known for performing best without unsupervised pre-training, but its derivative is always 0 when the input is less than 0, which can cause gradient backpropagation problems and result in some neurons being shut down permanently. As an improved version of ReLU, “LeakyReLU” overcomes this problem by allowing for smaller non-zero gradients, thereby improving the overall performance of the model.

It was found that without aligning the ECG with the PPG, the average value of Pearson’s *r* for 500 records was 0.977. This result demonstrates that the performance of the proposed W-Net model is second only to the performance of the DCT model using the TBME database. However, the data used in the DCT model are different from this study, and the results cannot be directly compared. Moreover, this study used 500 records, which is far more than the number of data used in the DCT model. Table 1 also shows that the phase error between the reconstructed ECG and the reference ECG is small. Better performance can be obtained by learning the PATs by the model itself rather than removing PATs in the preprocessing stage.

This study has some limitations.

1. Variations in PAT signals: The model proposed in this study is subject-specific, meaning that it captures the PAT of a specific individual during the training phase. Consequently, applying the model directly to multiple subjects presents a significant challenge due to variations in PATs between individuals, making the problem different and requiring the development of an inter-subject model.
2. Variability in PPG signals: PPG signals are susceptible to variability due to factors such as skin pigmentation, motion artifacts, and changes in blood volume. This variability can affect the accuracy of the reconstructed ECG signal.
3. Limited availability of training data: The availability of subject-specific training data for PPG-based ECG reconstruction is limited. This can make it difficult to train an accurate model that can generalize well to new subjects.

To address these challenges, the following recommendations can be made:

1. Data augmentation: Using data augmentation techniques can help mitigate the variability in PPG signals. Techniques such as adding noise, jittering, and randomizing the signal’s amplitude and frequency can increase the model’s robustness to signal variability.
2. Transfer learning: Transfer learning can help overcome the limited availability of training data by leveraging pre-trained models on similar tasks. For example, a pre-trained model on PPG-based heart rate estimation can be fine-tuned on the ECG reconstruction task.
3. Model optimization: Optimizing the model architecture and hyperparameters can reduce the computational requirements of the end-to-end model. Techniques such as pruning, quantization, and compression can reduce the model’s size and improve its efficiency.
4. Validation on large and diverse datasets: To ensure the model can generalize well to new subjects, it is crucial to validate its performance on a wide range of diverse datasets. This validation process can help uncover any biases in the model and ultimately improve its overall performance.
5. Deployment considerations: Considerations such as hardware requirements, power consumption, and real-time performance should be taken into account when deploying the model in real-world applications. For example, deploying the model on a mobile device with limited resources may require additional optimization techniques.

## 5. Conclusions

In conclusion, the PPG2ECGps algorithm, which is based on the W-Net architecture and is designed to be patient-specific, has shown promising results in the reconstruction of electrocardiogram (ECG) signals from photoplethysmography (PPG). The model’s ability to learn PAT information in long signal segments eliminates the phase error that is typically introduced during the preprocessing phase of aligning ECGs and PPGs based on feature points. The experimental results validate the effectiveness of the proposed model in reconstructing ECG signals that are highly similar to the reference ECG signals, with a small phase error.

Moving forward, the proposed model’s applicability can be further enhanced by generalizing it to multiple subjects. This will enable the model to be used in a wider range of settings, making it more practical and useful in real-world applications. Overall, this paper presents a promising approach to reconstructing ECG signals using PPGs, with the potential to significantly improve patient monitoring and diagnosis in the healthcare industry.

## Figures and Tables

**Table 1:** Comparison of the PPG2ECGps algorithm’s performance, with and without alignment of the reconstructed ECG with the reference ECG.

	Align ${\mathrm{ECG}}_{\mathrm{rec}}$ with ${\mathrm{ECG}}_{\mathrm{ref}}$	*RMSE* (mV)	*r*	$\bar{d}$ (mV)
Experiments I	No	0.037 ± 0.028	0.977 ± 0.029	0.010 ± 0.004
Experiments II	Yes	0.037 ± 0.027	0.978 ± 0.026	0.010 ± 0.004

**Table 2:** Evaluation of the subject-specific PPG2ECGps algorithm against other existing algorithms in the literature for reconstructing ECG signals from PPG signals. Note: NR stands for not reported. RMSE, *r*, and $\bar{d}$ stand for the root mean squared error, Pearson’s correlation coefficient, and the normalized dynamic time warping distance, respectively.

Method	Data Used	Alignment Required in Preprocessing	Segment Length	*RMSE* (mV)	*r*	$\bar{d}$ (mV)
DCT Model [18]	TBME-RR [36]: 42 Records MIMIC III [37]: 103 Records Self-collected: 2 Records	Yes	Beat	NR	0.984 0.940 0.904	NR
XDJDL model [19]	MIMIC III [37]: 33 Records	Yes	Beat	NR	0.88	NR
Bi-LSTM model [20]	MIMIC III [37]: 100 Records	Yes	1 s 2 s 3 s 4 s	0.063 0.068 0.063 0.059	0.893 0.874 0.891 0.904	NR
This study (PPG2ECGps)	Cuffless [25]: 500 Records	No	8.192 s	0.037	0.977	0.010

## Data Availability

The data can be downloaded via this link: https://archive.ics.uci.edu/ml/machine-learning-databases/00340/ (accessed on 1 January 2020). The source code will be made available upon request.

## Abbreviations

The following abbreviations are used in this manuscript:

PPG	photoplethysmography
ECG	electrocardiogram
PAT	pulse arrival time
